# Enhancing Engagement and Treatment Efficacy in Youth and Families with Persistent Trauma Exposure

**DOI:** 10.3390/children12121650

**Published:** 2025-12-05

**Authors:** Jana Pressley, Joseph Spinazzola, Irene Jun, Sophia N. Hamilton, Julian D. Ford, Richard Kagan

**Affiliations:** 1 Foundation Trust, Melrose, MA 02176, USA; jana@complextraumainstitute.com (J.P.); joseph@complextraumainstitute.com (J.S.); 2 School of Counseling, Richmont Graduate University, Atlanta, GA 30339, USA; 3 School of Social Work, Adelphi University, Garden City, NY 11530, USA; richardkagan7@gmail.com; 4 Rosary College of Arts, Education, and Sciences, Dominican University, River Forest, IL 60305, USA; shamilton@dom.edu; 5 Department of Psychiatry, University of Connecticut School of Medicine, Farmington, CT 06030, USA; jford@uchc.edu

**Keywords:** complex trauma, developmental trauma disorder, persistent trauma, predictability, preventability, assessment, engagement, treatment planning

## Abstract

Emerging research highlights important distinctions in symptomatology between Posttraumatic Stress Disorder (PTSD) resulting from a single, discrete event, complex interpersonal traumas in the past, and the pervasive effects of chronic, ongoing complex trauma. Despite these well-documented differences, much of the existing practice and professional guidelines for PTSD-focused interventions apply a uniform framework across the distinct clinical presentations resulting from different types and timing of trauma exposure. This gap carries significant clinical consequences, as individuals may be treated for PTSD and comorbid diagnoses or behavioral difficulties without recognition of the impact of persistent ongoing exposure to trauma. The present article is a clinical applications paper that directly builds upon a prior published theoretical and empirical literature review study that introduces the construct of persistent trauma. The objectives are to (1) examine the effects of persistent trauma; (2) explore four types of persistent trauma exposure differentiated by the predictability and preventability of past and current trauma; and (3) offer intervention strategies tailored to each type of persistent trauma. Using a composite case study methodology, we present intervention strategies to inform treatment for children and families who continue to experience each form of persistent trauma.

## 1. Introduction

Many useful treatment models have been developed to support youth and their families in recovering from traumatic experiences; however, the intervention strategies have typically focused on alleviating the symptoms of Posttraumatic Stress Disorder (PTSD) in the aftermath of singular traumatic events, with limited attention to differential clinical presentations that manifest from prolonged, recurrent exposure to trauma. In more recent years, there has been an increased recognition of the need for intervention models focused on complex trauma:

Complex trauma is best understood as a dual problem of (1) ongoing or recurrent exposure to interpersonal trauma typically originating in the context of a child’s caregiving system and (2) the consequent developmental deficits and emergence of emotional, social, cognitive and behavioral difficulties [1,2,3]. The most prevalent combination of risk factors involves exposure to some blend of impaired caregiving (e.g., due to parental substance abuse, mental illness, or intimate partner violence), neglect, emotional abuse, and physical abuse [4,5,6]. Additionally, complex trauma exposure intersects with other pervasive forms of life adversity including systemic racism, identity-based trauma, poverty, intergenerational trauma, and ancestral or historical trauma [7,8,9,10].

Complex trauma-informed intervention models have significantly expanded the lens of treatment, based on the assumption that early and chronic childhood traumatic experiences disrupt normative child development [11,12,13]. There are several complex trauma-oriented treatment models that offer clinicians specific techniques to integrate into practice including Attachment, Self-Regulation, and Competence [14], Integrative Treatment for Complex Trauma [15,16], Real Life Heroes: Resiliency-focused Therapy for Complex Trauma [17], Sensory Motor Arousal Regulation Therapy [18], Structured Psychotherapy for Adolescents Responding to Chronic Stress [19], Trauma-Adapted Family Connections [20], and Trauma Affect Regulation Guide for Education and Therapy [21]. In addition to recognizing classic PTSD symptoms, these intervention frameworks emphasize building or enhancing attachment relationships, developing self-regulation capacity, and supporting the growth of developmental competencies that may have been inhibited as a result of the deprivation, threat, harm, and losses caused by complex trauma [4]. Moreover, these treatment models recognize the added complexity of treatment that is the focus of this article, when exposure to complex trauma is not only in the past but is also ongoing in the present and into the future [22].

## 2. Persistent Trauma

Building upon notable advances in the treatment of children and youth exposed to complex trauma [11,23], Kagan and colleagues [24] introduced the concept of persistent trauma to capture trauma exposure that is ongoing or recurrent and at times unpredictable and unpreventable. Introducing a Multi-Dimensional Therapy Planning Guide for Complex Trauma (MTP) for complex trauma, these scholars delineated four categories of persistent trauma exposure: (1) ongoing, predictable and potentially preventable trauma exposure; (2) recurrent, predictable trauma exposure that is only partially preventable with periods of transient safety; (3) pervasive, unpredictable and unpreventable trauma exposure; and (4) past persistent trauma exposure that is not continuing in the present or likely in the future. Persistent trauma is associated with greater vulnerability, worse outcomes, limited access to resources, and the cooccurrence of complicating factors including systemic and structural traumas [24]. Elaborating on this seminal theoretical and empirical framework, the objective of the present paper is to inform clinical practice by describing the unique challenges posed when treating children and youth who are survivors of those four distinct forms of complex trauma and offer strategies for engagement and interventions.

### 2.1. Differential Intervention Strategies for Persistent Complex Trauma Exposure

The present paper outlines strategies in complex trauma intervention, guided by an understanding of the chronicity of trauma exposure. In other words, has the direct threat of danger abated, or is the client still dealing with ongoing peril? Utilizing the metaphor of navigating the ongoing storms of life, the following four categories outline the different types of trauma exposure chronicity to consider when formulating the treatment needs for families who are still facing danger and adversity that strike in different patterns, over different periods of time, and at varying levels of intensity.

#### 2.1.1. Storm Warnings: Ongoing Predictable & Preventable Complex Trauma Exposure with Persistent Complex PTSD/Developmental Trauma Disorder (DTD) Symptoms

Storm Warnings represent chronic relational trauma that co-occurs with an expectation of adversity. Safety gains may be possible with the development of supportive, protective practices. For these children and families, the storms are cyclical. There are often brief seasons of relative calm in which there might be a moment to stabilize, but with the knowledge that ominous clouds are often gathering on the horizon. With the threat of recurring adversity that is relatively predictable in nature, there is opportunity for treatment to build on existing strengths and resources in families and communities [1,25]. Treatment can enhance security by working collaboratively on strengthening relationships, coping skills and tangible safety planning practices with the child, parents/caregivers, extended family and the community [26,27,28].

#### 2.1.2. Living in the Path of the Storm: Ongoing Predictable & Unpreventable Complex Trauma Exposure & Persistent Complex PTSD/Developmental Trauma Disorder (DTD) Symptoms

This category parallels the previous one but is defined by ongoing, inherently unpreventable exposure to trauma that is built into family and community structures. Children and families with a history of recurring, predictable but unpreventable stressors may also experience transient periods of relative security. However, they may never settle into a sense of psychological or physical safety as there are continued risks and exposures to adversity embedded into the structure of their families and/or communities which may have been experienced over multiple generations [29,30,31]. For these clients, collaborative treatment must focus on discovering and enhancing what works to create transient security and to increase youth, family and community capacities for predicting, preventing and protecting themselves [26,32].

#### 2.1.3. The Endless Storm: Ongoing Unpredictable & Unpreventable (Pervasive) Complex Trauma Exposure & Persistent Complex PTSD/Developmental Trauma Disorder (DTD) Symptoms

In this group, there is both a history of considerable trauma and an ongoing exposure to adversity that is indefinite and without an expected end. Youth and families with past and present-day trauma exposure may be living with a history of ongoing, multigenerational trauma [9,33,34]. Trauma exposure tends to be unpredictable and chronic [8]. This is the most complicated trauma exposure and intervention category. Effective treatment requires validating the child and family’s experiences of chronic trauma and increasing the capacity to navigate relentless storm conditions and ongoing risks [22,24,27]. Further, treatment often involves strengthening or building essential relationships and coping skills to help youth and families increase relational security and resilience [35,36].

#### 2.1.4. Reliving the Storm: Past Complex Trauma Exposure & Persistent Complex PTSD/Developmental Trauma Disorder (DTD) Symptoms

Reliving the storm concerns the long-lasting impact of chronic complex trauma exposure and the continued experience of significant distress despite present safety and stability. For these children and families, the actual danger of the storm itself has dissipated, often following steps taken by parents/caregivers, extended families or communities to increase protection of the youth [26,32,36]. Nevertheless, youth or families may continue to experience the world as if they were living in the eye of a storm in survival mode with symptoms of Complex PTSD/DTD that interfere with optimal development [1,37].

For each of these categories of trauma chronicity, we present case studies to suggest and illustrate practices that can supplement evidence-supported trauma treatments and bridge the gaps between youth, caregivers, and clinicians that often lead to treatment ruptures. The primary objective of the present paper is to use case study methodology to illustrate strategies for delivering evidence-supported trauma treatment to youth and families affected by four different types of persistent trauma. Case vignettes are composites of our collective work with numerous children and families contending with these four categories of persistent trauma exposure and contain no protected health information or data from specific, identifiable individuals.

## 3. Composite Case Study Methodology

Design: A practice-based, multiple-case study was conducted to explore the four categories of trauma exposure chronicity and the applicability of intervention strategies centered on enhancing engagement and relational safety, developing self-regulation skills, and processing traumatic memories. Composite cases were authored and assigned to each category using theory-informed clinical judgment based on a client’s history of relational trauma, predictability and preventability of trauma exposure, ongoing exposure to adversity, and barriers to treatment.

Participants: A cross-case synthesis was conducted to identify recurrent patterns in (1) severity and chronicity of trauma exposure; (2) ecological and contextual circumstances; (3) clinical presentations; and (4) intervention strategies. We reviewed multiple real cases to construct four composite case vignettes that preserved these common elements while omitting any potentially identifying details. Individual consent was not required as these were fully de-identified composites drawn from multiple clients in the authors’ practices [38].

Procedure: The cases were derived from the clinical experiences of the authors working with children and families impacted by persistent trauma across a variety of service settings throughout the United States. Composite cases were constructed to map onto one of the four categories of persistent trauma exposure. A second author provided a peer review of these assignments and included their rationales. Once a consensus was reached, the composite cases were finalized.

## 4. Complex Trauma-Focused Intervention

Drawing on the guidelines of the National Child Traumatic Stress Network’s complex trauma workgroup, each of the categories of trauma exposure chronicity outlined above will be considered under the treatment components of (1) building engagement and relational safety; (2) self-regulation & psychoeducation around past, present & future danger; and (3) processing traumatic experiences and expanding resilience [1].

### 4.1. Building Engagement and Relational Safety

At any level of persisting trauma exposure, it is helpful for therapists to begin by providing psychoeducation to caregivers related to the various types of safety that are essential for healing (e.g., emotional, relational, and physical) then guide them in how to build and enhance safety in these areas over time [22,27,39]. Therapists model and support connection between youth and their caregivers, while assisting them in practicing new ways of interacting that validate youth’s experiences [40]. There are many existing intervention strategies that can guide caregivers in fostering attunement to their child’s needs and strengthening the relationship; however, the caregiver must simultaneously feel supported and understood by the therapist (and other adults in their life) in order to most effectively learn how to manage their own reactions to the ongoing process of healing and change [41,42]. For youth who lack safe, emotionally supportive and committed caregivers, we look for opportunities to strengthen existing caregiving relationships and cultivate new ones with appropriate mentors, community leaders, elders, and trained non-specialists where feasible [23,43].

### 4.2. Self-Regulation and Psychoeducation Around Past vs. Present Danger

Psychoeducation for youth around the stress response system and learning to distinguish their activated triggers is paramount, as they are learning to distinguish between past and present-day threats [8,23]. Therapists can support clients to cultivate awareness of their emotions, thoughts, and bodily sensations, and can teach youth and caregivers about concepts such as hyperarousal vs. hypoarousal [34,40,44]. It is equally important that caregivers are well informed about the slow and steady process of separating past and present triggers, as it will inevitably be confusing and taxing on the caregiver’s patience when their youth are demonstrating non-linear progress over time [27]. When caregivers are able to regulate and care for themselves in the midst of understanding the nature of complex trauma, they are best able to support the growing regulation capacity of their children [1,40]. This, in turn, can open up the possibility of living in the present and envisioning a future.

### 4.3. Processing Traumatic Experiences and Expanding Resilience

In the context of emergent relational security and regulation capacity, youth can begin to process traumatic memories [41]. Depending on the age and developmental stage of the youth, there are a variety of modalities that can facilitate such processing, including but not limited to: play therapy, sand tray therapy, EMDR, or other more structured models of developing a trauma narrative [23,45]. In the long term, the goal of successful complex trauma treatment is to guide the youth and families toward an identity that is beyond survival and adversity. Youth benefit from opportunities to explore their interests and passions and expand their identities beyond trauma history and treatment context [40]. As youth and families begin to heal, they can shift identity in a manner that is focused on engaging in a connected and meaningful life.

The treatment recommendation sections below are written using the language of youth and caregivers, which acknowledges the ideal that as clinicians we can engage and work with youth in the context of an adult support system as much as possible. However, we recognize that so many of our vulnerable clients are transition-age youth, falling in the developmental stage of older adolescence to young adulthood. These young people are often truly without family support, and treatment will need to be adapted to supporting the youth independently while striving to foster a possible social system that can support treatment goals over time [40,46,47].

## 5. Category 1: Storm Warnings: When the Storms Keep Coming

When working with youth and families in this and the following categories, it is of the utmost importance from the initial therapeutic engagement to demonstrate awareness and understanding around the reality of ongoing threats to emotional and physical safety [27]. For these clients, there is valid concern and hypervigilance around risk factors that reemerge regularly in the clients’ lives, and if clinicians are not honoring of these factors, they cannot be attuned or relevant in shaping treatment [22]. From the beginning of the treatment process, it is important that clinicians are exploring and naming ongoing, present-day trauma exposures so that treatment can be designed to be useful in distinguishing and addressing past trauma, while seeking to prevent or mitigate the severity of present/future stressors. Youth and families in this category have both a history of chronic relational trauma and an expectation of continuous adversity. However, safety in the midst of recurring trauma has the potential to be significantly enhanced by concrete safety planning, and the development of new supportive or protective practices by youth, parents/caregivers, extended family or the community [23,25]. Examples in this category may be:

- Children who have supervised or unsupervised visits with caregivers with whom there is a history of maltreatment or ongoing conflict or relational strain;
- Children with a loved one who is incarcerated;
- Children in residential juvenile justice or mental health placements;
- Families with increased vulnerability during predictable periods of financial strain or fluctuating demands on primary caregivers.

### 5.1. Building Engagement and Relational Safety

Treatment providers work to build upon the existing attachment connections, while supporting caregivers in learning to validate and comfort the youth in their care. Adult caregivers in this category of chronic adversity have often lived in such a heightened state of survival themselves that they have not developed the self-awareness or co-regulation skills to support their children [10,42,48]. Therapists can begin by providing psychoeducation for caregivers in understanding their child’s emotions and behaviors that fall in the category of triggered response to past trauma [39]. Additionally, whenever there is expected risk of ongoing trauma exposure, it is of critical importance to guide caregivers in the distinction between triggered response and present-day hypervigilance, so they are aware of how to respond accordingly [27].

In order to address the ongoing, predictable exposures to adversity, therapists can work with primary caregivers to brainstorm around safety planning. With support, families can put structures in place to distinguish between times of relative safety versus increased risk and can learn to address fears and support needs more explicitly [45]. When possible, it is of great value to draw upon the practical assistance of a client’s social support system, whether it be extended family and/or community resources that might enhance emotional and physical safety. Therapists can guide clients in brainstorming how to better access specific networks beyond the therapy office to address the tangible needs of the family (e.g., educational support, legal advocacy, food and housing support, financial assistance) [24,49,50]. Engaging clients in conversations about added reinforcements to safety can encourage a process of collaboration with community organizations, faith-based communities, or extended family when available, to expand the youth and family’s safety net in seasons of increased risk [39,43].

### 5.2. Self-Regulation & Self-Reflective Processing Around Past vs. Present Danger

Therapists work with youth directly to help identify coping skills and practices that are most useful for regulation when they are triggered by past trauma reminders [36]; likewise, providers validate the simultaneous existence of the hypervigilance that has served to support survival when danger presents itself [34]. For the youth who still needs to be able to activate self-protective instincts in the face of physical or psychological danger, providers should engage openly about honoring the role of the stress response system in keeping that youth safe in the scenarios in which ongoing risk exists [22]. Therapists can get practical and specific with youth to begin to distinguish safe versus unsafe places, people, and time periods in their lives, and support engaging new regulation strategies in those instances over time. For youth who have the active support and participation of caregivers in the therapeutic process, it is helpful to utilize activities and interventions that strengthen or build caregiver attunement and co-regulation. Supporting caregivers in differentiating between youth’s triggered reactions to past events and their responses to actual present danger is critical, as each requires a different intervention approach. For youth and families with known and predictable ongoing stressors, such as recurring mental health crises with a family member or visits with a triggering caregiver, therapists can help by:

- making explicit plans around how to prepare for the stressor ahead of time when possible
- identifying ways to actively cope in the midst of the anticipated event
- engaging in a recovery process after the stressor

Supporting youth in the development and practice of skills to better manage adversity can build a felt sense of success that is cumulative and confidence-building. As families work together in this process, it can also serve the purpose of enhancing trust between youth and their supporting caregivers [39].

### 5.3. Processing Traumatic Experiences and Expanding Resilience

Helping a client begin to process their experiences should go beyond the trauma narrative to include their larger life story. Such a story can ultimately include the challenges and periods of suffering, the various strategies of survival over the years, the strengths that have contributed to resilience in light of ongoing stressors, and possibilities for increasing those strengths over time to build a safer future [41]. For those who are still facing the storms gathering on the next horizon, any trauma processing must be approached with the caveat that those storm clouds exist. If a youth is able to begin sharing their story and still holds fear for the portions of their present life that are frightening or tenuous, the attuned therapist must hold that complexity and assist the client in piecing together their story in a manner that acknowledges the past trauma and the stressors yet to be faced [40].

### 5.4. Case Study

Danny is an 8-year-old, biracial boy who lives with his mother and 3 older siblings in a rural community a few miles outside of a mid-sized city. His mother, Tara, is an Indigenous American and his father, Michael, is Caucasian. Michael and Tara have been divorced for 3 years, and Tara has full custody; however, the kids still have supervised visitation 1–2 times/month with their father who is on court probation after serving a prison sentence for assault and battery. Prior to the divorce and arrest, Michael lived in the home and was verbally abusive to Tara. He was physically abusive to Danny and his siblings, although this was never substantiated as Michael would explain to the police when they were called that he has the right to physically discipline his children. This behavior was only taken seriously after the assault that occurred toward an adult peer in a local bar.

Michael often mentions his dream of shared custody in the future when spending time with the children, and Danny has repeatedly expressed to his mother that he gets scared when his father brings up this topic, begging her to not let his father take him. After visitation days, Danny’s mother reports that he will act out for several days at home and school, with both verbally and physically aggressive behavior. At home, Danny has recently started making disrespectful comments to his mother, blaming her for allowing his father to treat him and his siblings badly. He has begun to repeat some of the phrases that his father used to say to his mother, but he is now making these statements toward his mother and female peers in his class. Danny’s mother is perplexed because her “sweet little boy” on one hand appears terrified of being alone with his father, and on the other hand is beginning to mimic his behavior. After getting called in for a parent-teacher meeting at the school, Tara reaches out to a local community mental health agency in the nearby city to see if there is a therapist who will be able to help her manage Danny’s behavior.

Given Danny’s age, the therapist at the community mental health center scheduled an initial intake session with Tara to get a thorough developmental history, and to learn about the overall family and community context. The therapist explored Tara’s perspective of how the past traumatic experiences and ongoing contact with Michael have impacted Danny, as well as her other children. Although Tara always feels awkward and fears judgment in medical settings, she found the therapist to be warm and truly invested in understanding her concerns and hopes for Danny’s treatment. The therapist directly asked about Tara’s cultural identity and values and explored how Danny and his siblings identify in terms of the diverse heritage on both sides of their family. Tara has the opportunity to share the ways in which Danny’s early childhood years were entrenched in what she described as constant emotional upheaval, and how she often feels intense guilt for what she perceives as a failure to protect Danny from violence.

Danny was reluctant to be meeting a therapist, as a result of past encounters with case managers whom he experienced as forcing him to have supervised visitations with his father. His first impression however is that she seemed nice enough and made him feel good by telling him about the affection and pride that his mother shared when they talked about him. The therapist also informs him that although she is aware of some of his past experiences, she is not going to rush him into talking about those topics until he feels a bit more ready. Although this all feels like a relief and is better than Danny thought it would be, it still takes the next few weeks—and a lot of silence—before he begins opening up to the therapist. In exploring the contents of the therapist’s toys and tools on her shelf, Danny was eventually drawn to the basket of action figures and superheroes. The therapist was quiet for a few moments the first time he picked out a couple action figures and began playing out a physical altercation. As he experimented with getting a bit louder and more aggressive between the two characters, he was surprised that the therapist allowed him to demonstrate this conflict and did not immediately jump in to make him calmer like the other adults in his life had. Over time, the therapist would ask some questions about his characters and their conflict, and ultimately Danny was able to label himself and his father as the main characters. Over the next few weeks, Danny was gradually able to share some of his experiences and fears around his father, as well as his anger and confusion about his mother: “Why didn’t she protect me from him when he hit me?” “Why didn’t she put him in jail earlier?” “What is she going to do if the court makes me see him more?” As Danny was able to verbalize these questions—first to the therapist and eventually to his mother—they were able to start having hard conversations in therapy, which began to decrease the volatile reactions at home. In addition, the therapist worked with Danny’s mother to develop ways to respond differently, as she was dealing with her own trauma recovery.

Danny’s ongoing visitation schedule with his father needed to be addressed early in the work, as this was going to be an ongoing stressor for the foreseeable future. The therapist worked with Danny to start exploring what starts to happen to him in his thoughts, emotions, body sensations, and behaviors when he has an upcoming visit, and then they talked about those same reactions and what they look like after the visits. Danny and his therapist were able to identify action figures that helped him represent his before-, during-, and after-visitation reactions and they started working on a plan to help him learn how to better manage those reactions. They came up with tools that he could use, and eventually, he found a miniature action figure that he would carry in his pocket on visitations just to remind himself that he would be okay. Just knowing that the figure was in his pocket seemed to help him calm down and breathe when he started to panic. Danny’s therapist worked concurrently with his mother to understand these new resources, so that she could encourage his healthier coping at home.

Practically, a few strategies targeting the legal process were useful for Danny and his mother over the next few months of treatment. It was useful for Danny to learn—at an age-appropriate level—the timeline of his visitation and what his family rights were in the process of custody and visitation. Although his dad might be able to take his mother to court to request changes to custody, Danny came to understand that there were many steps involved, and that his mother and therapist would help him understand any possible changes well before they transpired. The increased commitment to transparency helped Danny’s hypervigilance settle a bit, although he found an action figure to represent the “always-there guard” who lived inside of him just in case there were any surprises.

Additionally, the therapist helped Danny notice that some of those fears were coming out in school in ways that were both getting him in trouble and making him lonely as his peers would stop wanting to spend time with him. Danny was actually quite funny, charming, and athletically skilled, and when he was able to start managing his outbursts with the tools he learned in therapy, his behavior at school began to improve and he learned to channel his intense feelings into sports and activities. Danny would continue to benefit from therapeutic support as he navigated the coming years moving into early adolescence—as his insight and reactions to his father evolved with maturity—with the foundation provided by this early phase of therapy.

## 6. Category 2: Living in the Path of the Storm with Transient Protection

This category of recurrent exposure builds on the previous category, with the distinction that the ongoing traumatic stressors are, by definition, not preventable, despite transient periods of safety. Those periods of safety are likely to always be shadowed by the inevitable return of danger and adversity. Examples in this category include:

- Children with safe and stable foster care, but with uncertainty about long-term relationships with caregivers or the potential return to previously neglectful or abusive homes or communities;
- Children who are living with a caregiver who was historically neglectful or abusive, but has been able to make sufficient changes to maintain custody;
- Children with a caregiver or sibling who is in and out of treatment for a mental or physical illness that has contributed to risks for family members;
- Children and young adults who have previously been gang-involved and continue to live in the community under the threat of peer/community violence, despite developing new ways of living;
- Children and families with complex trauma exposure who are continually dealing with the chronic adversity of poverty in their home and community, leaving them at risk for loss of support or resources that can come with little warning;
- Children and families with relatives who are living with life-threatening illnesses, with periods of time when the illness is contained or receding.

### 6.1. Building Engagement and Relational Safety

Children and families in this category may often describe themselves as “on guard” and hyperalert to real and present danger, which creates a constricted perspective of the world during moments when threat may not be present [10,51,52]. Initial and ongoing engagement with this population requires the utmost respect toward the ways that clients have survived and found coping strategies thus far, validating the ongoing risks and threats. Engagement and treatment efficacy can be enhanced by helping children, their caregivers, and relevant community support systems better understand the cycle of traumatic stressors [53]. This, in turn, can help children and families learn to better anticipate and prepare for seasons of adversity and engage in safety enhancing strategies with intentionality [24]. It is of critical importance to understand that caregivers of children in this category are also, by definition, living in the path of the storm, thereby requiring the treatment providers to engage with patience, humility, and empathy for the role these caregivers play in supporting their children in the midst of collective adversity [48]. Psychoeducation around the understandable reactions to the child and family’s triggered experience will be paramount, alongside support for caregivers to shift their responses and stance toward the children in their care to one that is increasingly calm and protective [27].

### 6.2. Self-Regulation & Self-Reflective Processing Around Past vs. Present Danger

Any discussion of building or enhancing regulation capacity with this population must be built on a foundation of honoring the survival strategies that have served the client thus far, while beginning to acknowledge practices that may not be sustainable long term [8,23]. The skills and distinction between past and present are similar in this category to those discussed under Storm Warnings, with the distinction that the storms may be less predictable and the calm seasons are less reliable than when living in the path of the persisting storm.

### 6.3. Processing Traumatic Experiences and Expanding Resilience

For children or families who are facing ongoing adversity, therapists can take advantage of periods of relative calm. These may occur on a predictable basis, for instance, when a grandparent is present or a chronically ill caregiver is experiencing a reprieve from symptoms. During these time periods, providers can help identify and build on existing children’s interests and talents, as well as family and cultural strengths, expanding possibilities for growth [9,54]. Pockets of transient safety can also provide opportunities to explore new interpersonal resources for the family. This can include identifying mentors [10], gleaning wisdom from the family’s cultural heritage and practices [55] or re-connecting to a relative or family friend who can be an added resource moving forward [33]. Caregivers who feel supported and respected by providers may engage in conversations about the resilience of past generations, introducing children to stories representing strength in the midst of adversity and offering insight into their family heritage—a part of themselves that they may not have had the opportunity to learn about previously [43].

Providers can work with children, their caregivers, and their cultural communities to reflect upon and reinforce a strength-oriented identity and cultural heritage, honoring the resilience that has sustained them thus far. Developing a life narrative that denotes individual and community resources, protective factors and competency in the midst of adversity is encouraged. Building on a perspective of time—extending from the past into the future—can also help enable children and parents/caregivers to elicit connections with previous generations and strengths in their cultural heritage [54,56]. Life story work can bring out memories of being nurtured by a parent from earlier years, the love of a grandparent, or a child’s hope for caring for their own family in the future. Safe place or safe relationship imagery of caring relationships can be enhanced through creative arts (e.g., drawing, music, movement) and other sensory-motor activities [40]. The aim of this is to help children identify and solidify their values and build a sense of identity, even in the path of the storm.

### 6.4. Case Study

Carlos Almeida is an 11-year-old, Hispanic boy who lives with his biological father and stepmother, after the death of his mother to cancer when he was 5. Carlos is one of 5 children in the home, with 2 older brothers (ages 14 and 16) and 2 younger half-sisters (ages 1.5 and 4). Prior to his mother’s cancer diagnosis, Carlos’s father was physically and verbally abusive toward her and the boys, which mostly happened when he was intoxicated. He got sober during his wife’s treatment which remained until after her death, with a few episodes of relapse in recent years. In the present, he drinks intermittently and has periodically become physically threatening toward Carlos and the younger girls; however, when this happens now, the older boys will step in and there will be physical altercations that escalate to a severe level of violence. The police have been involved a few times now, with dad spending time in jail and Carlos’s oldest brother going to juvenile detention for a couple months. Carlos’s stepmother is someone who he describes as kind, but both she and his father work long hours, often leaving him to babysit his young sisters. The Almeida family lives in low-income housing, and Carlos reports being nervous when he walks to and from school, as he is often approached by his older cousins and their friends, who are believed to be gang-involved. Carlos has witnessed 2 incidents of community violence leading to death in the past year and has not yet told anyone that he often throws up after arriving home from school, due to the symptoms of anxiety. Carlos struggles to sleep at night, panicked by sounds inside and outside the house—as he has become vigilant both to the sound of his father’s voice when he comes home from work, as well as the sounds of rising conflict in the courtyard below his 2nd story apartment window. When he does get to sleep, he often awakes to nightmares and cold sweats, leaving him exhausted and often falling asleep in class during the day. Carlos has begun to get identified as “checked out” at school due to his sleepiness, and he does not know how to begin to tell his teachers why he is unable to stay awake in class. Carlos was referred to treatment by his school social worker, and his father has expressed willingness to bring him to sessions and attend his own meetings to support his sobriety. However, Mr. Almeida’s family expresses skepticism regarding his commitment to sobriety.

When Carlos, his father, and stepmother first enter the clinic, Carlos expresses to his parents that it feels “stupid and pointless” to be there. He spots a couple of young Caucasian staff members coming in and out of the lobby who do not look like they would relate to life in his neighborhood. He feels the same shut down hopelessness that often arises when he is at school. When the therapist meets with Carlos and his parents, however, he actually asks questions and makes statements in response that feel like he might be interested in things that are important to Carlos. Carlos notices that the therapist is not immediately blaming him for his behavior, being harsh with his parents, or even trying to convince him to be more positive, which is something that some of his teachers will do. Carlos is surprised, as he has had to participate in mandated family therapy sessions before related to his father’s and brothers’ trouble, and as the first few sessions of treatment continue, this therapist seems to be more of a real person, who is also realistic about the actual scary and uncertain circumstances of his life. The therapist will meet with him and his parents—mostly separately and occasionally together—and has been teaching everyone about trauma and linking Carlos’s struggles (depression, anxiety, intrusive thoughts and memories, school problems) to the past experiences in his family. Carlos observes that his father has not stopped coming to the sessions yet, and he suspects it is because this therapist is somehow able to talk to him about his past aggression and his drinking as a problem, but with kindness and without judgment. While learning about the impact of the past, the therapist also seems to “get it” when talking to Carlos that many of the past issues could happen again if his brother gets aggressive and ends up back in trouble, if his dad drinks again, and if something else happens because there are many risks in his neighborhood that are not going away soon. The most helpful thing Carlos’s therapist does is teach him to pause and ask himself if a panicky feeling is about the past or something that is going on right now.

There are a few practical strategies that the therapist helps the Almeida family implement:

- The family identified an aunt whom Carlos trusts and is able to accompany him to school in the morning, and they also found an afterschool program where he can spend time for a couple of hours after school until his stepmother can bring him home. This has significantly decreased his day-to-day anxiety and physical reactivity related to his fear of community trauma exposures.
- For days when things go as planned, his family was able to get him an inexpensive phone through a community safety program which has helped him feel more confident that he can call for help if needed.
- After disclosing his fear about the sounds of violence outside his window at night, Carlos rearranged his bedroom with the bed being as far away as possible from the window, and his parents found him a white noise application on his phone that can mute the sounds that were triggering his fear and keeping him awake.
- Being better rested and more aware of the reason behind his sleepiness/shutdown at school is helping him be more engaged, and with the prompting and collaboration of his outpatient therapist, he has been able to use his school counselor for support when he is getting upset at school.

Once he started feeling a bit more settled in his daily life, Carlos and his therapist started processing some of his memories and grief around the loss of his mom when he was younger, as well as the memories of domestic violence in the same childhood era. Additionally, since Carlos’s parents have been participating in his therapy, his father has remained committed to sobriety. Separately in parent check-in sessions, the therapist has been able to work with Mr. Almeida in reflecting on his own past trauma and the ways that his drinking and aggression were patterns developed from his own childhood survival repertoire. Carlos is able to use his individual time with his therapist to remain cautiously hopeful for the best but prepared to get support should he need to handle a relapse on his father’s part.

## 7. Category 3: The Endless Storm

Children in this category have not only already endured extensive traumatic exposure, but they also continue to live in circumstances in which they continue to be endangered with no foreseeable end. When working with children and families for whom the chronic trauma exposure persists in day-to-day life, professionals are often at a loss to know how to best intervene, given that symptoms continue and are exacerbated by re-traumatization. Treatment models that do not help these children to maintain necessary vigilance may be grossly misattuned to the reality of these individuals’ lives and be experienced as invalidating how the child and family are coping with real ongoing threats and harm. This can lead to clients not showing up for appointments, not allowing home visits or dropping out of treatment. Therapists can promote engagement and treatment efficacy by recognizing both vulnerabilities and possibilities for these children and parents/caregivers beginning with validation of ongoing risks and injuries [24]. Examples in this category include:

- Children and families living with violence, neglect, emotional, physical, or sexual abuse, addiction, threats of abandonment or self-harm by family members that have not been validated or where steps cannot be taken to increase prevention or promote healing;
- Children and families who live with chronic exposure to severe community violence, necessitating ongoing awareness of actual physical risk and loss;
- Children in placement programs who have been told by parents or authorities that they will be returning to homes where they experienced neglect, emotional, physical or sexual abuse, threats of abandonment or family violence;
- Children (and transitional age young adults) who are living with continuous adversity in the absence of an obvious support network;
- Children and families with ongoing exposure to racial, cultural, or identity-based discrimination, hate crimes, institutional betrayal, or religious persecution;
- Children and families dealing with the ongoing fears, losses, and uncertainty due to their immigration status;
- Children and families dealing with the stressors related to chronic poverty, homelessness, or a lack of other essential resources.

### 7.1. Building Engagement and Relational Safety

Moving toward greater safety and stability entails increasing safe, emotionally supportive relationships. The challenges of engaging caregivers or creating stronger connections for trauma-impacted children are even more profound in this category of trauma exposure chronicity, because everyone directly involved is likely to be highly impacted and symptomatic [26,42]. Therefore, relational engagement for these children and parents/caregivers can often benefit from casting a wide net within and outside of the family to provide a multidisciplinary professional and peer support system for the care management necessary to address their complex unmet needs.

### 7.2. Self-Regulation & Self-Reflective Processing Around Past vs. Present Danger

For individuals and families living under such circumstances, there may be limited opportunity to decrease hypervigilance, as the heightened awareness of danger remains necessary for survival. In such cases, clinicians would be doing children and caregivers a grave disservice if they are not attuning to the persisting threats to safety. Rather, it is important to be working with children and their caregivers to increase awareness of their stress response system and normalize understandable reactions that emerge in crisis moments. Children living in the endless storm are often referred because of high-risk behaviors that have led to harm to the children or others [49,51]. The predominant focus on behavioral change or narrowly on skill development in many systems of care can make it harder to uncover the fears driving high-risk behaviors, as well as complicate efforts to foster relationships that could promote safety and growth [24,36]. With these realities in mind, therapists should seek to both understand the high-risk behaviors through the frame of survival and simultaneously brainstorm progressively safer alternatives to support regulation.

### 7.3. Processing Traumatic Experiences and Expanding Resilience

For children and families in the midst of pervasive traumatic stress, processing trauma is complicated because the trauma is ongoing and significant. Processing in the way that we classically consider with symptoms of PTSD is not a predominant goal, as children are still very much in “survival mode” [23]. However, if a child is feeling overwhelmed or paralyzed by a particularly intrusive memory that is getting in the way of adaptive functioning, there may still be value in processing specific experiences. Naming and identifying the past and present traumatic exposures also provide significant value, for the purpose of differentiating past and present threat, and brainstorming how to continue to utilize the safest and most effective survival-based coping mechanisms to optimize safety.

Stories of navigating intergenerational trauma, historical trauma, ongoing racism and oppression can be a powerful component of a life narrative for children who have access to caregivers who can provide such insight and reflection. This includes learning from older generations and community groups who have grappled with risks or threats faced by the child including historical traumas, marginalization and microaggressions linked to race, religion, sexual orientation, gender identity, immigration status or ethnic origin [33,57]. Activities that explore learning from the past can help create increased safety for children and parents/caregivers to move beyond the limits of living in daily survival mode [54]. Such a focus can help children and families hold the complexity of acknowledging and grieving traumas and losses, naming the ways they have attempted to cope in the past (even when the strategies were not optimal), while also growing stronger together in solidarity around the strengths they can draw upon in ongoing adversity [29,31,58]. This is a “both/and” approach to trauma processing, in which we are helping clients and families identify and validate adverse experiences and survival adaptations, while also avoiding the pitfall of asking them to process trauma that still remains tenuous with realistic threat of danger or risk factors reemerging.

### 7.4. Case Study

Ava is a 17-year-old African American female who lives with her grandmother, who was recently diagnosed with Parkinson’s disease. She moved out of her biological mother’s home to live with an aunt after an incident at the age of 12 in which her mother’s boyfriend propositioned her for sex, and her mother did not believe her when she told her. This was not the first time that she had been approached in such a manner, but it was the last straw when she told her mother and was accused of lying for attention. In reality, this proposition triggered memories and panic due to sexual abuse she experienced between the ages of 5–7 by another one of her mother’s male partners.

Ava spent a few years living with her aunt Tina, and although Tina was willing to allow her to stay, Tina’s emotional dysregulation due to her own mental health struggles often left Ava feeling unsafe in the home environment. Ava’s grandmother was someone who had been a resource in her life earlier in childhood, but she rarely saw her as she lived on the outskirts of the city, and it took 45 min to get to her apartment by train. When she became desperate enough at the age of 15, Ava applied to transfer to the school in her grandmother’s district, forged the necessary documents, packed her belongings, and showed up at her grandmother’s apartment to live with her. Although her grandmother took her in, she was increasingly showing signs of weakness and changes in personality, which ultimately resulted in her Parkinson’s diagnosis. Simultaneously, Ava began to experience bullying at school and racial discrimination in the community, which has traditionally been a white, working-class neighborhood. Ava was fired from her job at the grocery store after being falsely accused of stealing merchandise and struggled to get another job after this incident. A year after her move to her grandmother’s community, she was sexually assaulted in an abandoned building close to her school by 2 male classmates, whose identities she chose not to reveal due to her assumption that they would deny or blame her. Left with the triggering option of going to school and seeing them every day, Ava stopped attending school altogether 2 months into her junior year. Ava had a couple of friends from her old neighborhood who she stayed in touch with, but she was mostly isolated in her grandmother’s town. Her grandmother’s health seemed to be declining, and it was the nurse at her grandmother’s recent medical appointment who referred Ava to consider speaking with the social worker on staff, after she noticed that Ava looked pale and underweight compared to earlier weeks. Ava declined and shut down, but reconsidered in the coming weeks when she found herself dealing with intrusive thoughts about suicide. Most recently, Ava has learned that her grandmother is moving into a nursing facility, and Ava is forced to move back to her old neighborhood. She is able to use the spare room of her maternal aunt and uncle but will need to work to contribute to the rent and groceries. She feels lost, hopeless, and panicked given the state of danger she recalls that seems to still be accurate in her family neighborhood.

When Ava returns to her old neighborhood, she ambivalently takes the advice of the social worker at her grandmother’s medical clinic and calls a local mental health clinic to make an appointment. Ava is 17 years old by the time she begins therapy, which affords her the right to sign her own treatment consent in the state in which she lives. She is glad about this, as her aunt and uncle would not be supportive if they knew she was seeking therapy. The community-based clinic has a funded treatment scholarship program for which she is eligible, and she begins out of a state of desperation. Ava spends her first several weeks of therapy struggling to find the language to articulate the story of her life, which is compounded by the practical anxiety of needing to find a job without a high school degree. Her therapist works hard to balance addressing the time sensitive pragmatic survival needs while creating space for Ava’s severe level of depression and passive suicidality that she has disclosed. Ava’s level of physiological distress and paralysis make it complicated for her to present herself in a manner that is appealing to employers, so she and the therapist begin by learning how her depression and anxiety are manifesting in her body and building some simple day-to-day soothing skills that begin to calm her nervous system.

As Ava builds trust with her therapist, she is able to share more of her history of life growing up with her mother, her early adolescent years with Tina, and her transition in and out of her grandmother’s home. Ava does not have contact with her mother—a fact for which she holds tremendous guilt—and she worries constantly about her grandmother’s care in the nursing facility. When her therapist comments about the ways in which she carries the weight of the world on her shoulders, she breaks down in tears and also feels validated and understood.

There are a few practical strategies that the therapist helps Ava explore and implement over the coming months:

- With the therapist’s steady support and focus on social skills related to the job search, Ava is able to obtain employment at the front desk of a local boys and girls club, which is accessible to public transportation from her apartment.
- The center where she is receiving therapy offers a trauma-informed yoga class, which Ava ambivalently agrees to try with the patient encouragement of her therapist. When she begins, Ava finds that she benefits from the growing awareness of the tension she holds in her body—and this proves to be enlightening when she and her therapist eventually begin to explore her history of sexual trauma.
- The combination of yoga class and initial exploration of sexual trauma history highlights the fact that Ava is living in daily fear and hypervigilance when walking anywhere in the city. She feels keyed up and panicky in her body and often spirals into a sense of helplessness and fear that she would not be able to defend herself if attacked again. Ava’s therapist begins to explore the combined physical and cognitive processes associated with her abuse/assault history and helps her look into self-defense training. Through a friendly contact at the boys and girls club where she works, she is able to find a female-led martial arts gym with affordable rates where she can begin to learn strategies to feel stronger and more powerful in her body.
- It becomes clear to the therapist early on in the therapeutic process that Ava is highly intelligent and talented, and she begins to explore with Ava ideas around seeking her General Education Development in order to move forward in possible career exploration.

Over time, Ava learns to discuss her past and present-day life through the lens of what her therapist calls complex trauma and so many of her experiences make sense to her. She is currently trying to decide if she is going to re-initiate contact with her mother, an action that she knows could carry interpersonal risks to her stability. She has begun to visit her grandmother as often as she can on weekends, and as she has stabilized in her own mental health, she is able to spend connected time with her grandmother and enjoy meaningful conversations on her grandmother’s “good days.” Ava learns stories about her grandmother’s past trauma and resilience that explain why she moved to the suburb where she rebuilt her life, and these stories empower her to continue her own journey of education and future job exploration, despite the challenges she continues to navigate in her daily life.

## 8. Category 4: Reliving the Storm

In this category, we address the needs of children who have endured childhood, chronic relational trauma, but for whom exposure has ceased. For example, children who have been adopted into what is objectively an emotionally and physically safe home but continue to demonstrate significant distress. These children and the adults caring for them likely still need years of support to work through the impact of childhood exposure, as the effects on development related to DTD typically present long-term based on the chronicity and severity of early exposure [2,4,14]. For these children, symptoms and life struggles are based in survival-based adaptation rooted in earlier years, resurrected by present-day triggers that bring back reminders of the past and activate behavioral and relational patterns of coping [4,11].

### 8.1. Building Engagement and Relational Safety

Although treatment can be slow and tedious, caregivers who are dedicated to understanding and intervening in children’s recovery can participate in building nurturing attachment relationships, while supporting the growing regulation capacity and healthy functioning of trauma-impacted children. When working with children and families falling in this category, therapists begin by providing psychoeducation to caregivers related to the various types of safety that will be essential for healing—emotional, relational, and physical—guiding caregivers in how to build and enhance safety in these areas over time. Therapists also model and support connection between children and their caregivers, while assisting them in practicing new ways of interacting that validate the child’s experiences. The caregiver must simultaneously feel supported and understood by the therapist (and other adults in their life) in order to most effectively learn to manage their own reactions to the ongoing process of healing and change.

### 8.2. Self-Regulation & Self-Reflective Processing Around Past vs. Present Danger

The assumption for clients in this category is that they have the capacity to grow and can begin to dedicate internal resources toward recovery, rather than survival, because they are out of harm’s way. However, children may not yet identify with this capacity because they do not have a frame of reference for feeling emotionally or physically safe. Psychoeducation for children about their body’s stress reactions and learning to notice their activated triggers is of the utmost importance, as the child learns to live in a world without active threats. Therapists teach children and caregivers about concepts such as hyperarousal vs. hypoarousal, which also provide insight about the complicated process of separating past traumas from present reminders. When caregivers are able to regulate and care for themselves in the midst of understanding the nature of complex trauma, they are best able to support the growing regulation capacity of their children and increase relational security. For children and caregivers alike, building or strengthening emotionally supportive relationships and regulation skills can provide the foundation for the re-integration of traumatic memories [14,21,27].

### 8.3. Processing Traumatic Experiences and Expanding Resilience

As children in this category begin to stabilize and experience growth in their coping capacity, there is increased emotional, cognitive and interpersonal space to engage in new relationships, skills, and competencies [37]. As these skills continue to increase, children are gradually able to process the past traumatic experiences, particularly within a system that has learned how to safely hear and respond to their story. Helping children to develop a life narrative—which includes but is not limited to their trauma stories—is highly recommended, as this can begin to expand children’s perspectives on potential strengths and resources that have existed in their past and present [27,40,56].

### 8.4. Case Study

Frannie is a 12-year-old, Caucasian female who has been adopted in the past year. Frannie was born into a chaotic biological home and spent the first 3 years of her life in emotionally and physically impoverished conditions. Frannie’s father left prior to her birth, and her mother was severely depressed and dealing with significant drug addiction. Frannie was removed from the home after a neighbor discovered her crying and home alone in the apartment on more than one occasion, and she was placed in the temporary care of her maternal grandparents. Frannie’s grandparents took her in reluctantly as they were already raising the two teenage sons of their adult son who was incarcerated. When Frannie was 5, they gave up guardianship after it was reported by a kindergarten teacher that there was suspicion of physical and sexual abuse by Frannie’s cousins in the home. Frannie’s grandparents decided they could no longer keep her safe in the home with her cousins, with the added burden of being on a fixed income with their own health problems. It was at this time that Frannie entered the foster care system, and eventually her mother worked with the agency to make an adoption plan, acknowledging that her challenges were too overwhelming to her capacity to parent. At this time, Frannie’s mother had become sober and was maintaining sobriety but decided it was in the best interest of her daughter, herself and other family members that Frannie grow up in another family.

Throughout her early and school age years, Frannie would get quickly triggered into inconsolable tantrums which left new caregivers feeling helpless and overwhelmed, leading to a series of failed placements and school transitions. By 5th grade, she was reading 3 grade levels behind and cycling out of her 5th foster home when she met the Thompson family. Mr. and Mrs. Thompson were patient and caring foster parents who had previously adopted an adolescent who was now in college. They took the risk of taking on a longer-term foster placement with Frannie. After a slow and rocky period of getting to know Frannie and her interpersonal and emotional patterns, the Thompsons began to develop a level of trust with her. When Frannie turned 11, the Thompsons made the commitment to adopt Frannie and seek treatment for her and consultation for themselves.

Although Frannie’s adoptive parents had some experience with parenting a child with a trauma history, the intensity of her dysregulation and the focus on her adoptive caregivers as the target of her panic and rage when she perceived rejection, were new to them. During the intake session, the therapist thoroughly explored the patterns of Frannie’s emotional and behavioral dysregulation and reviewed the extensive records of Frannie’s Child Protective Services file which outlined early childhood trauma exposures and available details related to her foster care placements and disruptions. She was able to frame Frannie’s symptoms through the lens of DTD which was a new concept for them and enabled them to better understand her extreme interpersonal reactions, her cognitive delays, and her regressive behaviors when triggered.

The therapist began working with Frannie individually and was able to negotiate an arrangement in which Frannie agreed they could connect with her adoptive parents in the last 15 min of most sessions to help them better support her needs at home and/or discuss any pressing communication issues in their relationship. Frannie quickly and easily connected with the therapist at a surface level, but it became clear that she would often present in a manner to look pleasing and compliant to the therapist, even though she struggled to manage the perceived threats of rejection at home with her adoptive parents. Frannie had learned over the years from her experience that treatment providers have the power to move you out of your home and even though this was not technically true in the current situation, it still felt true for Frannie. She and the therapist had to work together for some time to develop more of an authentic relationship in which she could express her true worries or frustrations without tempering with a fawn response.

The therapist worked steadily with Frannie and her adoptive parents—separately and together—to pinpoint her relational triggers that were ultimately leading to her outbursts at home, then to build co-regulation and self-regulation capacities. Frannie would not only get triggered by interpersonal cues that activated her fears (e.g., body language, tone of voice), but she would also be activated by the fear of abandonment and home displacement when her awareness of her own outburst had set in. Caregivers needed to learn to intervene as proactively and preventatively as possible at the initial trigger level, then work to soothe and reassure her of her safety and permanence in their family in the aftermath of explosions. Concurrently, the therapist worked directly with Frannie to understand and catch her own responses as early as possible, and to practice skills to regulate herself.

When Frannie got to the point of being sufficiently regulated to feel safe in her daily life, she was able to engage in processing some of the past traumatic experiences. Through a combination of sand tray therapy and EMDR, Frannie was able to work through some of the more intrusive memories of her earlier childhood traumatic exposures and frightening, abusive foster care placements. In time, Frannie’s system started to settle, and she began to make friends in her school.

Although Frannie and her adoptive parents stabilized considerably in the first year of therapy, Frannie’s treatment needs shifted when she turned 14 and landed more solidly in adolescence. With dating opportunities and sexual impulses emerging, a new chapter in therapy emerges. Despite the gains made in therapy, her adoptive parents were distressed upon learning that she had begun to dabble in risky relational behavior online with older boys and men who are giving her the attention she still so desperately craves. The groundwork laid earlier in treatment predicted this type of possibility in such a manner that the parents were able to recognize the behavior not as an afront to their nurturing, but as a continuous struggle against the intense need for emotional and physical care beyond what they can even give, due to her profound level of early neglect.

Although this case reflects a client for whom active traumatic exposure is not present, it would be shortsighted to assume that Frannie will not face a variety of internal struggles related to her adoption and her biological family of origin. Traumatic grief related to the loss of connection to her mother and extended family is a critical factor that will present differently at various stages of development across Frannie’s life. Given that she lived with her biological family until the age of 5, she would likely have enough memories and awareness of their presence in her early life, and as she gets older, she may naturally begin to wonder and ask questions about her mother who gave up parental rights. If she knows or recalls the stories of her mother’s early addiction and battle to get sober and her grandparents health problems, she might worry about them and mourn the ability to have access to them. As Frannie continues to explore her identity over time, questions about her biological roots in both curious and anxious ways might emerge for her: “Are some of my interests and talents inherited from my biological parents? Will I develop a mental illness or become addicted to substances like my family of origin?” The questions and losses that can surface for children like Frannie are important to hold in mind as their own form of trauma complexity, even when the storms have seemingly settled externally.

## 9. Discussion

Throughout this paper we identified four categories of persistent trauma exposure and their relevant treatment considerations: storm warnings (ongoing, predictable, and preventable trauma exposures), living in the path of the storm (ongoing, predictable, but unpreventable exposure), the endless storm (ongoing, unpredictable, and unpreventable exposure), and reliving the storm (continuing to live as if one is in the eye of the storm in the absence of ongoing exposure). We incorporated a case study within each category to demonstrate how the nature of trauma chronicity affects the client’s functioning and how treatment can be adapted accordingly.

For storm warnings, Danny benefited from psychoeducation and anticipatory planning before, during, and after visitations, which demonstrates how reframing hypervigilance as a normative response to tremendous stress can open up space for identifying and utilizing coping strategies. At the living in the path of the storm level, Carlos demonstrates how windows of transient security can be further extended through concrete environmental modifications, improvements in the family structure, and community resources, which in turn, create opportunities to develop resilience and practice self-regulation skills. In the endless storm, Ava underscores the value of individualized care that is responsive to both her treatment needs and her immediate, practical needs. A holistic approach that integrates her trauma history, treatment barriers, current adversities, and future goals—while prioritizing safety and stabilization—highlights the functional gains that are achievable when care is guided by a framework of persistent trauma exposure. Finally, through reliving the storm, Frannie illustrates how relational safety with her parents, coupled with the gradual processing of traumatic memories brought awareness to and mitigated some of her survival-driven activations, to help her adjust to a life that is no longer organized around trauma.

All four case studies suggest that engagement is a key driver of treatment retention in the face of persistent current or past adversity. Engagement fosters trust in the clinician and helps establish a robust therapeutic alliance which propels treatment into skills development and memory processing. Beyond engagement, complex trauma-informed intervention with persistent trauma exposure entails that clinicians (1) provide explicit validation of ongoing risks and present symptoms; (2) identify specific triggers and coping skills to alleviate distress; and (3) build caregivers’ co-regulation skills. It should be emphasized that sociopolitical contexts are heavily embedded in the clinical presentations of persistent trauma exposure. Historical trauma, intergenerational trauma, systemic racism, armed conflict, and other forms of oppression are not background variables; they are active determinants of clients’ exposure to danger, access to care, and mental health outcomes. Situating care within these contextual factors encourages the clinician to integrate other safety nets into the clients’ lives and tailor treatment to be culturally sensitive, emotionally attuned, and responsive to their specific needs.

## 10. Limitations and Future Directions

Although effective intervention practices are presented, this paper is not without its limitations. First, the qualitative nature and reliance on illustrative vignettes limit generalizability and causal inference. The suggested strategies should be validated in future empirical research including program evaluations and treatment outcome studies. Subsequent research should complement qualitative insights with quantitative data through validated diagnostic measures to examine the association between these intervention strategies and changes in symptoms. Second, because these case studies did not employ validated outcome measures at standardized time points (baseline, mid-treatment, post-treatment, follow-up), we cannot confirm diagnoses, quantify domain-specific changes, or identify mechanisms of change. While the children described in the case studies would meet criteria for multiple diagnoses including PTSD, complex PTSD, and depression, we intentionally omitted diagnostic information to offer a humanistic, strengths-based, and non-stigmatizing approach to partnering with children and families who have been impacted by ongoing adversity to empower them to be active participants in their recovery. The present paper does not describe a full course of treatment for each case study, but rather provides general strategies on fostering engagement, relational safety, self-regulation, and resilience. Thus, conclusions about which treatment components contributed to improvements cannot be determined. Future studies should operationalize and test intervention strategies that target clinical, behavioral, and functioning outcomes relevant to persistent trauma. Third, inequity remains to be an ongoing problem. In certain parts of the United States and around the world, access to mental health providers may be limited or unavailable, particularly in rural and remote communities or areas stricken with poverty. Similarly, disadvantaged and marginalized children and families remain underrepresented in outcome studies due to structural barriers (e.g., unemployment, unstable housing, transportation) that hinder engagement and lead to treatment dropout. Future research should identify which environmental resources (e.g., perceived safety, transportation, family supports) and resource-navigation strategies most effectively increase treatment initiation, attendance, and retention. Finally, we recognize that the present paper does not sufficiently address specific considerations for vulnerable and historically marginalized populations, including Indigenous/Native and refugee communities, which merits more in-depth exploration in future research. All these limitations should be considered as we seek to establish an evidence base for the intervention strategies, reduce barriers to treatment, and promote more equitable access to care.

## 11. Conclusions

When working with children and families who are exposed to complex trauma, treatment outcomes may improve as clinicians explicitly differentiate the types of trauma chronicity, address the impact of persistent ongoing trauma, and subsequently select intervention strategies to target specific domains of functioning. Maintaining a consistent “both/and” stance of validating “symptoms” as reflections of understandable distress and as survival adaptations while fostering self-regulation, relational security, developmental competencies, and resilience is essential for sustaining engagement in treatment and preventing treatment ruptures. It is important to note that there will be fluidity between these categories for children receiving treatment. Conditions can change for families in both positive or negative directions based on factors outside of anyone’s control, and as clinicians it is important that we are always assessing for the impact of such change in our client’s healing process. When working with children and families through a complex trauma lens, and particularly with those for whom trauma exposure and adversity persist, there are key assessment and treatment foci to bear in mind:

- From the beginning stages of assessment and treatment, acknowledging immediate needs/priorities and how treatment can help with critical needs;
- Identifying immediate dangers and enhancing physical, emotional, and relational safety;
- Distinguishing adaptive responses from symptomatic reactions;
- Identifying accessible and reliable attachment persons and support systems;
- Developing a life narrative that includes traumatic adversities but focuses on self-capacities and protective/facilitative resources;
- Co-envisioning an immediate future that provides security and is self-affirming;
- Co-envisioning a long-term future that provides paths to agency and achievement.

With these principles as a guide, treatment can support by children and families as they navigate the pervasive storms created by persistent and ongoing complex trauma.

## Data Availability

The original contributions presented in the study are included in the article/Supplementary Material, further inquiries can be directed to the corresponding author.

## Supplementary Materials

The following information can be downloaded at: https://www.complextrauma.org/wp-content/uploads/2025/12/MTP-Guide.pdf (accessed on 27 November 2025).

## Abbreviations

The following abbreviations are used in this manuscript:

PTSD	Posttraumatic Stress Disorder
MTP	Multi-Dimensional Therapy Planning Guide for Complex Trauma
DTD	Developmental Trauma Disorder
EMDR	Eye Movement Desensitization and Reprocessing

## References

[1] Cook A., Spinazzola J., Ford J., Lanktree C., Blaustein M., Cloitre M., DeRosa R., Hubbard R., Kagan R., Liautaud J. (2005). Complex Trauma in Children and Adolescents. Psychiatr. Ann..

[2] D’Andrea W., Ford J., Stolbach B., Spinazzola J., Van Der Kolk B.A. (2012). Understanding Interpersonal Trauma in Children: Why We Need a Developmentally Appropriate Trauma Diagnosis. Am. J. Orthopsychiatry.

[3] Klasen F., Gehrke J., Metzner F., Blotevogel M., Okello J. (2013). Complex Trauma Symptoms in Former Ugandan Child Soldiers. J. Aggress. Maltreat. Trauma.

[4] Ford J.D. (2021). Why We Need a Developmentally Appropriate Trauma Diagnosis for Children: A 10-Year Update on Developmental Trauma Disorder. J. Child Adolesc. Trauma.

[5] Lanktree C.B., Briere J., Godbout N., Hodges M., Chen K., Trimm L., Adams B., Maida C.A., Freed W. (2012). Treating Multitraumatized, Socially Marginalized Children: Results of a Naturalistic Treatment Outcome Study. J. Aggress. Maltreat. Trauma.

[6] Spinazzola J., Hodgdon H., Liang L.-J., Ford J.D., Layne C.M., Pynoos R., Briggs E.C., Stolbach B., Kisiel C.L. (2014). Unseen Wounds: The Contribution of Psychological Maltreatment to Child and Adolescent Mental Health and Risk Outcomes. Psychol. Trauma.

[7] Allwood M., Ghafoori B., Salgado C., Slobodin O., Kreither J., Waelde L.C., Larrondo P., Ramos N. (2022). Identity-Based Hate and Violence as Trauma: Current Research, Clinical Implications, and Advocacy in a Globally Connected World. J. Trauma. Stress.

[8] Eagle G., Kaminer D. (2013). Continuous Traumatic Stress: Expanding the Lexicon of Traumatic Stress. Peace Confl..

[9] Fortuna L.R., Tobón A.L., Anglero Y.L., Postlethwaite A., Porche M.V., Rothe E.M. (2022). Focusing on Racial, Historical and Intergenerational Trauma, and Resilience. Child Adolesc. Psychiatr. Clin. N. Am..

[10] Pressley J., Smith R. (2017). No Ordinary Life: Complex Narratives of Trauma and Resilience in under-Resourced Communities. J. Aggress. Maltreat. Trauma.

[11] Briere J., Lanktree C.B. (2011). Treating Complex Trauma in Adolescents and Young Adults.

[12] Spinazzola J., Van Der Kolk B., Ford J.D. (2021). Developmental Trauma Disorder: A Legacy of Attachment Trauma in Victimized Children. J. Trauma. Stress.

[13] Stolbach B.C., Minshew R., Rompala V., Dominguez R.Z., Gazibara T., Finke R. (2013). Complex Trauma Exposure and Symptoms in Urban Traumatized Children: A Preliminary Test of Proposed Criteria for Developmental Trauma Disorder. J. Trauma. Stress.

[14] Blaustein M.E., Kinniburgh K.M. (2018). Treating Traumatic Stress in Children and Adolescents: How to Foster Resilience Through Attachment, Self-Regulation, and Competency.

[15] Briere J., Lanktree C.B. Integrative Treatment of Complex Trauma for Adolescents (ITCT-A) Treatment Guide, 2nd ed. https://keck2.usc.edu/adolescent-trauma-training-center/wp-content/uploads/sites/169/2016/06/ITCT-A-TreatmentGuide-2ndEdition-rev20131106.pdf.

[16] Briere J., Lanktree C.B. Treating Substance Use Issues in Traumatized Adolescents and Young Adults: Key Principles and Components. https://keck2.usc.edu/adolescent-trauma-training-center/wp-content/uploads/sites/169/2016/06/Treating-Substance-Use-Issues-20131106.pdf.

[17] Kagan R. (2016). Real Life Heroes: Toolkit for Treating Traumatic Stress in Children and Families.

[18] Warner E. (2020). Transforming Trauma in Children and Adolescents: An Embodied Approach to Somatic Regulation, Trauma Processing, and Attachment-Building.

[19] Habib M., Labruna V., Newman J. (2013). Complex Histories and Complex Presentations: Implementation of a Manually-Guided Group Treatment for Traumatized Adolescents. J. Fam. Violence.

[20] Collins K.S., Strieder F.H., DePanfilis D., Clarkson Freeman P.A., Tabor M., Linde L., Greenberg P. (2011). Trauma Adapted Family Connections: Reducing Developmental and Complex Trauma Symptomatology to Prevent Child Abuse and Neglect. Child Welfare.

[21] Ford J.D., Courtois C.A. (2020). Trauma Affect Regulation: Guide for Education and Therapy (TARGET). Treating Complex Traumatic Stress Disorders in Adults: Scientific Foundations and Therapeutic Models.

[22] Diamond G.M., Lipsitz J.D., Hoffman Y. (2013). Nonpathological Response to Ongoing Traumatic Stress. Peace Confl..

[23] Ford J.D., Courtois C.A. (2015). Treating Complex Traumatic Stress Disorders in Children and Adolescents: Scientific Foundations and Therapeutic Models, Reprint ed..

[24] Kagan R., Pressley J., Espinoza R., Lanktree C., Henry J., Knoverek A., Duffy S., Labruna V., Habib M., Blaustein M.E. (2022). Development of a Differential Assessment Guide to Improve Engagement with Youths & Families Living with Chronic Trauma. J. Child Adolesc. Trauma.

[25] Haine-Schlagel R., Walsh N.E. (2015). A Review of Parent Participation Engagement in Child and Family Mental Health Treatment. Clin. Child Fam. Psychol. Rev..

[26] Kiser L.J., Medoff D.R., Black M.M. (2010). The Role of Family Processes in Childhood Traumatic Stress Reactions for Youths Living in Urban Poverty. Traumatology.

[27] Murray L.K., Cohen J.A., Mannarino A.P. (2013). Trauma-Focused Cognitive Behavioral Therapy for Youth Who Experience Continuous Traumatic Exposure. Peace Confl..

[28] Stevens G., Eagle G., Kaminer D., Higson-Smith C. (2013). Continuous Traumatic Stress: Conceptual Conversations in Contexts of Global Conflict, Violence and Trauma. Peace Confl..

[29] DeGruy J. (2005). Post Traumatic Slave Syndrome: America’s Legacy of Enduring Injury and Healing.

[30] Lehrner A., Yehuda R. (2018). Cultural Trauma and Epigenetic Inheritance. Dev. Psychopathol..

[31] Mullan J. (2023). Decolonizing Therapy: Oppression, Historical Trauma, and Politicizing Your Practice.

[32] Brody G.H., Yu T., Beach S.R.H. (2016). Resilience to Adversity and the Early Origins of Disease. Dev. Psychopathol..

[33] Menakem R. (2017). My Grandmother’s Hands: Racialized Trauma and the Pathway to Mending Our Hearts and Bodies.

[34] Smith J.R., Patton D.U. (2016). Posttraumatic Stress Symptoms in Context: Examining Trauma Responses to Violent Exposures and Homicide Death among Black Males in Urban Neighborhoods. Am. J. Orthopsychiatry.

[35] Bryant R.A. (2023). Attachment Processes in Posttraumatic Stress Disorder: A Review of Mechanisms to Advance Theories and Treatments. Clin. Psychol. Rev..

[36] Correia-Santos P., Sousa B., Ford J.D., Maia Â.C., Pinto R.J. (2024). Resilience in the Aftermath of Trauma: Classes of Adjustment in at-Risk Youth. Psychol. Trauma.

[37] Burke Harris N. (2018). The Deepest Well: Healing the Long-Term Effects of Childhood Adversity.

[38] Yin R.K. (2017). Case Study Research and Applications: Design and Methods.

[39] Gopalan G., Goldstein L., Klingenstein K., Sicher C., Blake C., McKay M.M. (2010). Engaging Families into Child Mental Health Treatment: Updates and Special Considerations. J. Can. Acad. Child Adolesc. Psychiatry.

[40] Ford J.D. (2021). Progress and Limitations in the Treatment of Complex PTSD and Developmental Trauma Disorder. Curr. Treat. Options Psych..

[41] Amaya-Jackson L., DeRosa R.R. (2007). Treatment Considerations for Clinicians in Applying Evidence-based Practice to Complex Presentations in Child Trauma. J. Trauma. Stress.

[42] Evans G.W., Kim P. (2013). Childhood Poverty, Chronic Stress, Self-Regulation, and Coping. Child Dev. Perspect..

[43] Becker K.D., Boustani M., Gellatly R., Chorpita B.F. (2018). Forty Years of Engagement Research in Children’s Mental Health Services: Multidimensional Measurement and Practice Elements. J. Clin. Child Adolesc. Psychol..

[44] Teicher M.H., Samson J.A. (2016). Annual Research Review: Enduring Neurobiological Effects of Childhood Abuse and Neglect. J. Child Psychol. Psychiatry.

[45] Yim S.H., Lorenz H., Salkovskis P. (2024). The Effectiveness and Feasibility of Psychological Interventions for Populations under Ongoing Threat: A Systematic Review. Trauma Violence Abus..

[46] Blaustein M.E., Kinniburgh K.M. When Age Doesn’t Match Stage: Challenges and Considerations in Services for Transition-Age Youth with Histories of Developmental Trauma. https://www.pathwaysrtc.pdx.edu/focal-point-S1506.

[47] Spinelli T.R., Riley T.J., St. Jean N.E., Ellis J.D., Bogard J.E., Kisiel C.L. (2020). Transition Age Youth (TAY) Needs Assessment: Feedback from TAY and Providers Regarding TAY Services, Resources, and Training. Child Welf..

[48] Ford J.D., Courtois C.A. (2020). Treating Complex Traumatic Stress Disorders in Adults: Scientific Foundations and Therapeutic Models.

[49] Clarke A.T., Grassetti S.N., Brumley L., Ross K.Y., Erdly C., Richter S., Brown E.R., Pole M. (2023). Integrating Trauma-Informed Services in out-of-School Time Programs to Mitigate the Impact of Community Gun Violence on Youth Mental Health. J. Prev. Interv. Community.

[50] Hansen M.C., Small L.A., Ghafoori B., Campbell C. (2024). Understanding Treatment Attitudes in the Path towards Trauma Recovery in Urban-Dwelling Trauma-Exposed Latinx Adults. J. Ethn. Cult. Divers. Soc. Work.

[51] Lanktree C., Briere J. (2015). Treating Complex Trauma in Children and Their Families: An Integrative Approach.

[52] Pressley J., Spinazzola J. (2018). The Developmental Consequences of Exposure to Complex Trauma in Childhood and Adolescence. Violence and Trauma in the Lives of Children. Volume One: Overview of Exposure.

[53] Lawrence T.I., Hong J.S., Sopchak K.S., Voisin D.R. (2023). The Association between Exposure to Community Violence and Somatic Symptoms through Bullying Victimization among African American Adolescents in Chicago: A Developmental Trauma Approach. J. Clin. Psychol..

[54] Ortega-Williams A., Beltrán R., Schultz K., Ru-Glo Henderson Z., Colón L., Teyra C. (2021). An Integrated Historical Trauma and Posttraumatic Growth Framework: A Cross-Cultural Exploration. J. Trauma Dissociation.

[55] Hicks Peterson T. (2024). Practicing Liberation: Transformative Strategies for Collective Healing and Systems Change: Reflections on Burnout, Trauma and Building Communities of Care in Social Justice Work.

[56] Serpeloni F., Narrog J.A., Gonçalves De Assis S., Quintes Avanci J., Carleial S., Koebach A. (2021). Narrative Exposure Therapy versus Treatment as Usual in a Sample of Trauma Survivors Who Live under Ongoing Threat of Violence in Rio de Janeiro, Brazil: Study Protocol for a Randomised Controlled Trial. Trials.

[57] Brave Heart M.Y.H., DeBruyn L.M. (1998). The American Indian Holocaust: Healing Historical Unresolved Grief. Am. Indian Alsk. Native Ment. Health Res..

[58] Gutiérrez N.Y. (2022). The Pain We Carry: Healing from Complex PTSD for People of Color.

